# Solvent-driven modulation of phenolic composition and biofunctional activities of three *Mentha aquatica* formulations: integrated in vitro and in silico insights

**DOI:** 10.1186/s40643-026-01093-6

**Published:** 2026-07-06

**Authors:** Meryem Tourabi, Amira Metouekel, Mohamed Jeddi, Mohamed Chebaibi, Nesrine Benkhaira, Kawtar Fikri-Benbrahim, Youssouf Ali Younous, Turki M. Dawoud, Esmael M. Alyami, Hina Ali, Gehan M. Elossaily, Badiaa Lyoussi, Elhoussine Derwich

**Affiliations:** 1 Laboratory of Biotechnology, Conservation and Valorization of Bioresources, Faculty of Sciences, Sidi Mohamed Ben Abdellah University, Fez, Morocco; 2 Sorbonne Universités, Université de Technologie de Compiègne, EA 4297 TIMR, Compiègne, Cedex 60205 France; 3 Microbial Biotechnology and Bioactive Molecules Laboratory, Faculty of Science and Techniques, Sidi Mohamed Ben Abdellah University, P.O. Box 2202, Road of Imouzzer, Fez, Morocco; 4 Ministry of Health and Social Protection, Higher Institute of Nursing Professions and Health Techniques, Fez, Morocco; 5 Biomedical and Translational Research Laboratory, Faculty of Medicine and Pharmacy of Fez, Sidi Mohamed Ben Abdellah University, Fez, 30000 Morocco; 6 Evangelical College, BP 1200, N’Djamena, Chad; 7 Department of Botany and Microbiology, College of Science, King Saud University, P.O. BOX 2455, Riyadh, 11451 Saudi Arabia; 8 Shanghai Key Laboratory for Molecular Engineering of Chiral Drugs, School of Chemistry and Chemical Engineering, Shanghai Jiao Tong University, Shanghai, 200240 People’s Republic of China; 9 Research Centre for Advanced Materials Science (RCAMS), King Khalid University, Abha, 61413 Saudi Arabia; 10 Department of Basic Medical Sciences, ge of Medicine, AlMaarefa University, Diriyah, Riyadh, 13713 Saudi Arabia; 11 Research Center, Deanship of Scientific Research and Post-Graduate Studies, AlMaarefa University, Diriyah, Riyadh, 13713 Saudi Arabia; 12 Unity of GC/MS and GC-FID, City of Innovation, Sidi Mohamed Ben Abdellah University, Fez, Morocco

**Keywords:** *Mentha aquatica*, HPLC-ESI-FULL-MS *in-silico* approach, Solvent polarity, Antioxidant activity, Antibacterial activity

## Abstract

The goal of this work was to examine the effect of different solvents (Water, EtOH 70%, and acetone) on the phenolic composition, antioxidant, and antibacterial capacity of Moroccan *Mentha aquatica* L. leaf extract. To this end, HPLC-ESI-FULL-MS was used to characterize the extracts, while the Folin-Ciocalteu and aluminum trichloride techniques were used to evaluate the total phenolic and flavonoid contents. To assess the antibacterial capacity, the microdilution technique was performed to calculate the minimal inhibition concentration (MIC), and minimal bactericidal concentration (MBC). Phytochemical profiling revealed that the extracts were rich in bioactive constituents, particularly ferulic acid derivative, caffeoyl-protocatechuic acid derivative, quercetin, and diosmetin 7-O-beta-D-glucuronide. The hydroethanolic extract contained the highest levels of total phenolic (62.2 ± 1.2 mg GAE/g DW) and flavonoid (29.15 ± 0.09 mg QE/g DW) contents, exceeding those of the acetonic extract (22.2 ± 0.6 and 10.17 ± 0.07 mg GAE/g DW, respectively) and the water extract (22.4 ± 0.6 and 10.9 ± 0.6 mg QE/g DW, respectively). This extract also showed the strongest antioxidant effect, recording an IC_50_ of 0.060 ± 0.001 mg/mL in the DPPH assay, and an EC_50_ of 80 µg/mL in the RP test. In addition, it shows a great total antioxidant capacity, reaching 75.1 ± 2.0 mg EAA/g DW when compared to water and acetonic extracts (28.5 ± 1.4 and 21.1 ± 0.1 mg EAA/g DW, respectively). The antibacterial potential ranges from 0.78 ± 0.05 mg/mL to 12.6 mg/mL. In-silico prediction highlighted diosmetin 7-O-beta-D-glucuronide, quercetin, and equisetumpyrone as the key contributors to antioxidant capacity, while quercetin, 2,3,8-Tri-O-methylellagic acid, and diosmetin 7-O-beta-D-glucuronide were involved in antibacterial activity.

**Graphical Abstract:**

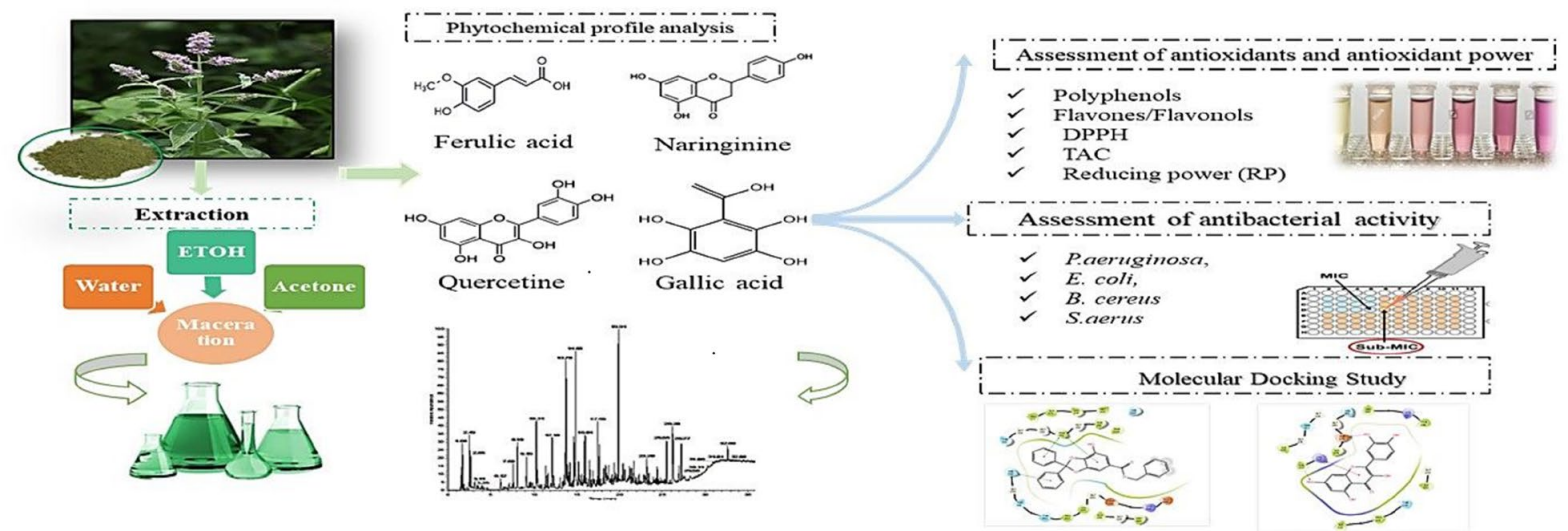

## Introduction

The genus *Mentha* (Lamiaceae) comprises around 25 to 30 mint species distributed across nearly every continent, thriving in diverse ecological conditions (Tourabi et al. 2023c). The *Mentha* genus is widely valued for both culinary and therapeutic purposes, largely due to its richness in bioactive phytochemicals. Species of the genus are reported to possess a wide range of medicinal applications, including antibacterial, anti-inflammatory, antidiabetic, anti-ulcer, biocidal, antiemetic, antispasmodic, anti-allergic, tonic, and antihypertensive activities. (Shaikh 2014). These varied pharmacological properties could be attributed to the presence of their abundant secondary metabolites, like carotenoids, terpenoids, phenolic acids, flavonoids, fatty acids, tannins, and saponins.(Park et al. 2019).

Water mint (*Mentha aquatica* L.) is an annual herbaceous plant species with a widespread distribution occurring across nearly every continent except Antarctica and South America. In traditional medicine it has been extensively used across various cultures for the treatment of a wide array of diseases (Esmaeili et al. 2006). More specifically, leaf infusions of *M. aquatica* are used orally in digestive complaints, fever, headache, and gingivitis as mouthwashes (Thi et al. 2020). In Algerian medicine, the tea of *M. aquatica* is used to treat coughs and digestive disorders(Nadjib Chaker et al. 2014), reflecting antispasmodic and carminative effects. It is also used in traditional South African medicine for colds (Esmaeili et al. 2006), and respiratory disorders (Olsen et al. 2008), consistent with expectorant and anti-inflammatory activities. Their essential oil is used as a stimulant and astringent (Esmaeili et al. 2006), indicating tonic and tissue-contracting effects. Moreover, *M. aquatica* is traditionally used to treat, menstrual disorders, arthritic diseases, rheumatism, and supports analgesic mechanisms. It’s used in depression (Asadollah-Pour et al. 2021), suggesting sedative or CNS-modulating properties. It is also tacken as a tonic reported analgesic, relaxing,, and sedative properties (Gruenwald et al. 2000). Research has well-established several pharmacological properties of *M. aquatica*, including insecticidal, which were verified by both fumigation tests and repellency tests (Tourabi and Baghouz 2024), antihemolytic properties, were confirmed by erythrocyte protection assays (Ebrahimzadeh et al. 2010), as well as antimicrobial effecency, (Mkaddem et al. 2009). There were also hepatoprotective properties that were validated in models that mimic liver damage (Pereira et al. 2019), and anticancer effects were confirmed by cytotoxic and antiproliferative assays (Sharma et al. 2014). Additionally, gastroprotective effects were verified in experimental models of gastric damage (Gul et al. 2015), while antiemetic capacity was verified by in vivo experiments (Gruenwald et al. 2000). The delve into the phytochemical analysis of *M. aquatica* identified a range of rosmarinic, cinnamic, ferulic, gallic, and ellagic acids, as well as catechin, chlorogenic acid, quercetin, naringenin, rutin, and hesperidin (Pereira et al. 2019). The abundance and diversity of these compounds are considered responsible for the species’ pharmacological properties, which may vary according to ecological and environmental factors.

The choice of solvent has a significant impact on the effectiveness of extracting these bioactive substances since solvent polarity influences solubility and selectivity toward particular phytochemicals (Tungmunnithum et al. 2018). Frequently employed solvents comprise hexane, chloroform, dichloromethane, ethyl acetate, acetone, water, and alcohols (ethanol, methanol, and butanol) used either individually or in combination (Iloki-Assanga et al. 2015). The solubility of various phytochemical classes can be affected by the polarity of the extraction solvent, which has an impact on the final extract’s composition. Components are preferentially solubilized by increasingly polar solvents (hexane < chloroform < acetone < ethyl acetate < ethanol < distilled water), while nonpolar substances are extracted by less polar solvents, while polar compounds, like flavonoids and phenolics, are more effectively extracted by polar solvents (Wakeel et al. 2019; Nawaz et al. 2020). Therefore, Solvent properties influence the yield and composition of phytochemicals, which in turn influence the bioactive properties (Tourabi et al. 2023b, 2025).

Several previous studies have employed solvents, namely methanol, ethanol, chloroform, acetone, and water, for the recovery of bioactive substances from species within the *Mentha* genus (Jurić et al. 2021; Tourabi et al. 2023b). However, most of these research studies only focus on the general efficiency of extraction instead of investigating the specific influence of solvent polarity on the selective extraction of bioactive compounds. In fact, recent investigations of the *Mentha* genus have suggested innovative perspectives in using green extraction technologies with a focus on using eco-friendly solvents with high yields of bioactivity. Despite these recent achievements in extraction technologies, no specific study exists that evaluates the effect of different solvent polarities on the selective extraction of bioactive compounds from *M. aquatica* leaves in Morocco and compares their antioxidant and antibacterial potential using green solvents. This study aims to evaluate the effect of different solvent polarities (acetone, ethanol 70%, and distilled water) on the recovery of bioactive compounds from *M. aquatica* leaves, as well as their antioxidant capacity and antibacterial activity. By linking solvent polarity to both bioactive compound yield and functional properties, this work seeks to identify the most effective solvent for maximizing bioactive recovery while aligning with sustainable extraction trends in plant bioactive research.

## Materials and methods

### Botanical material

In June 2021, *M*. *aquatica* leaves were gathered from Merja Zerga, also referred to as Moulay Bousselham Lagoon in Morocco, and produced as herbarium samples. The identification of these specimens was confirmed by Professor Amina Bari, a botanist from Sidi Mohamed Ben Abdellah University’s Department of Biology. The faculty herbarium is home to the voucher specimen, which is identified by the designation 002MAMZ2121.

### Preparation of extracts

Different solvents were used to obtain various extracts, and the extraction process was carried out following the method described elsewhere (Tourabi et al. 2023b). The *M. aquatica* leaves were dried by air drying, which was done in the shade at room temperature ranging from 22 °C to 25 °C until a constant weight. The leaves were ground and sieved to obtain a particle size of approximately 0.5–1 mm.

To prepare the extracts, 1 g of dried plant powder was mixed with 10 mL of each solvent (hexane (P′ = 0.1), chloroform P′ = (4.1), ethyl acetate (P′ = 4.4), acetone (P′ = 5.1), 70% ethanol (P′ = 6.3), and distilled water (P′ = 9.0)) and macerated at room temperature for 7 days with mechanical agitation. All extractions were conducted in amber glass containers to minimize light exposure and were sealed to limit oxygen contact. The mixtures were then filtered through Whatman No. 1 (Millipore, Burlington, Massachusetts (MA), USA) paper, and the filtrates were concentrated at 40 °C using a rotary vacuum evaporator (Büchi Labortechnik, R-210 B-490, Flawil, Switzerland). The resulting crude extracts were stored in amber glass at − 20 °C until further use.

### Screening and selection of extracts

Following extraction, all solvent extracts were subjected to preliminary screening for antioxidant, polyphenol, and flavonoid content. Based on these results, the most potent extracts (water, acetone, and 70% ethanol) were selected for detailed analysis of antioxidant, phenolic compounds, and content, and antibacterial activity.

### Estimating the yield of extraction

The filtrate was concentrated using a Büchi R-210 rotary evaporator under reduced pressure at 40 °C to obtain the crude extract. The extraction yield was then determined as a percentage, calculated according to the following formula (1).

1 $$\mathrm Y\;_{\mathrm{extract}}\;(\%)\;=\;({\mathrm W}_{\mathrm{ext}}\;(\mathrm g)/{\mathrm W}_{\mathrm{plant}}\;(\mathrm g))\ast100$$

*Y* extract%: extraction yield presented in percentage; *W*_*ext*_: Weight of dry extract; *W*_*plant*_: Weight of dry plant.

### HPLC-ESI-FULL-MS analysis

The analytical method used was adapted from the protocol developed by Metouekel et al. (Metouekel et al. 2024) for the characterization of *Cannabis sativa* seed extracts, with slight modifications to suit our own matrix, namely *Mentha aquatica* extracts. Before injection, the samples were passed through a disposable syringe filter with a 0.45 μm pore size (Millex^®^-GP, 0.45 μm pore size). The chromatographic analysis was carried out on a DIONEX UltiMate 3000 UHPLC+ system (Thermo Fisher Scientific) equipped with a diode-array detector (DAD), and a high-resolution electrospray ionization quadrupole-Orbitrap™ mass spectrometer (Exactive™ Plus Orbitrap, Thermo Scientific), covering a mass range of 50–6000 m/z. Data acquisition and processing were carried out using the Thermo Scientific Chromeleon CDS (Xcalibur Roadmap, version 4.1). Chromatographic separation was achieved on a Hypersil™ BDS C18 column (150 mm × 4.6 mm, 5 μm; Thermo Fisher Scientific, Waltham, MA, USA). The mobile phases consisted of (A) water acidified with citric acid (pH adjusted to 3.1) and (B) methanol. Gradient elution was applied over a 45-minute run, optimized for differential compound polarity. Mass detection was conducted in negative ion mode. Detected compounds were tentatively identified by comparison against the NIST MS Search 2.3 mass spectral library and MS/MS databases. While this method is still under refinement for complete validation, it has provided reliable preliminary chemical profiles of the *Mentha aquatica* extracts.

### Assessment of total phenolic content

The quantification of total phenolic compounds was carried out using the Folin–Ciocalteu method as described by (Tourabi et al. 2023a). Briefly, 50 µL each of the samples was mixed with 500 µL of Folin solution (0.2 N), then left for 5 min before 400 µL of 75 g/L Na_2_CO_3_ was added, followed by 2 h incubation in the dark. The absorbance reading was taken at 760 nm with a reagent blank containing all the reagents used in the analytical procedure without the sample or analyte, but the extract was replaced with the appropriate solvent. A calibration curve was prepared with gallic acid standards (0.016-2 mg/mL, R² = 0.996). The results for total phenolic content (TPC) were expressed as milligrams of gallic acid equivalents per gram of plant dry weight (mg GAE/g DW). Each experiment was carried out three times.

### Assessment of total flavonoid content

The total flavonoid content (TFC) was measured using the colorimetric method described by (Tourabi et al. 2023a). For this, 500 µL of AlCl₃ reagent solution (10%) was mixed with 500 µL of the plant extracts and standard solutions. After incubating at room temperature in the dark for one hour, the absorbance was read at 420 nm using a blank solution containing all reagents used in the analytical method, apart from the sample or the analyte. A quercetin standard curve (0.008-1.00 mg/mL; R² = 0.994) was used for calibration, and the results are expressed as milligrams of quercetin equivalents per gram of dry weight (mg QE/g DW). All experiments were performed in triplicate.

### In vitro antioxidant capabilities

The antioxidant potential of the *M. aquatica* leaf solvent extract was evaluated using three different assays: total antioxidant capacity (TAC), reducing power (RP), and 2,2-diphenyl-1-picrylhydrazyl (DPPH•) radical scavenging (Tourabi et al. 2023c). All measurements were conducted using a PerkinElmer Lambda 40 UV-VIS spectrophotometer (PerkinElmer Inc., Waltham, MA, USA).

The inhibitory concentration (IC₅₀) was determined from the inhibition curve, using BHT as a positive control, and expressed in mg/mL. Extracts were tested over a concentration range from 2 mg/mL to 0.003 mg/mL, with at least ten concentrations in total. The IC₅₀ values were calculated using nonlinear regression analysis, fitting the data to an appropriate model, and were derived from the sigmoidal dose-response curve. DPPH radical scavenging activity was calculated as a percentage of inhibition using the formula (2):

2 $$PI\;(\%)\;=\;\left(\frac{A\;control\;-\;A\;sample}{A\;control}\right)\times100$$

Where PI: Percentage of inhibition; A_control_: Absorbance of control; A_sample_: Absorbance of sample.

For the reducing power assay, ascorbic acid was used as a reference. The half-maximal effective concentration (EC₅₀) was determined from the absorbance graph (Y = ax + b; Y = 0.5) and expressed in µg/mL. Test samples were applied over a range from 2 mg/mL to 0.003 mg/mL, with a minimum of ten concentrations. The EC_50_ values were computed by employing nonlinear regression analysis. The EC_50_ is obtained from the curve. The results were expressed in terms of EC_50_, which is the extract concentration that elicits a response in 50% of the maximal response. The EC_50_ values were expressed in µg/mL.

The total antioxidant capacity (TAC) was measured with the use of an ammonium phosphomolybdate assay, as was done according to the method described by Prieto et al., (Prieto et al. 1999). The ascorbic acid served as the standard within a concentration range of 0.00048 to 1 mg/mL (R² = 0.9991). The results are expressed as mg of ascorbic acid equivalent per gram of dry weight (mg AAE/g DW). All measurements were done in triplicate.

### Assessment of antibacterial capacity

#### Microbial testing

The antibacterial activity of the selected solvent extracts was evaluated by microdilution against multiresistant strains: *Staphylococcus aureus* (ATCC 29213), *Escherichia coli* (25922), *Bacillus cereus* (6633), and *Pseudomonas aeruginosa* (27853). Mueller-Hinton broth was used for susceptibility testing, and nutrient agar for maintaining the cultures.

#### Evaluation of the minimal inhibitory concentration (MIC)

The MIC of different *M. aquatica* leaf extracts (water, 70% ethanol, and acetone) was determined using the microdilution method in sterile 96-well microplates, following CLSI guidelines with slight modifications (Fadipe et al. 2015). Briefly, bacterial suspensions were prepared from fresh cultures and adjusted to 0.5 McFarland (≈ 1 × 10⁸ CFU/mL), then diluted in Mueller–Hinton broth (MHB) to obtain a final inoculum of approximately 5 × 10^8^ CFU/mL per well.

Extracts were first diluted in 2% dimethyl sulfoxide (DMSO) to prepare a range of concentrations. The microplates were incubated at 37 °C for 18–24 h. After incubation, 10 µL of 0.015% resazurin solution was added to each well and incubated for an additional 2 h at room temperature. A color change from purple to pink indicated microbial growth, whereas the absence of color change indicated growth inhibition. The following controls were included in each assay: (i) a growth control consisting of inoculated MHB without extract, (ii) a sterility control containing MHB only, (iii) a vehicle control containing MHB, bacterial inoculum, and DMSO at the same final concentration used in the test wells, and (iv) positive controls using reference antibiotics (gentamicin and kanamycin). All experiments were performed in triplicate.

#### Evaluation of minimum bactericidal concentration (MBC)

To determine the minimum bactericidal concentration (MBC), 3 µL from each well that showed no visible bacterial growth were plated onto MH agar and incubated at 37 °C for 24 h (Namdev, D. et al. 2026).

### Molecular docking study

Molecular docking analyses were performed using Maestro 11.5 from the Schrödinger suite. This study aimed to investigate how the phenolic compounds from various solvent extracts of *M. aquatica* interact with the active sites of target proteins, including NADPH oxidase, beta-ketoacyl-[acyl carrier protein] synthase from *Escherichia coli*, and nucleoside diphosphate kinase from *Staphylococcus aureus*. The research focused on assessing both the antioxidant potential and the antibacterial activity of these phenolic compounds.

#### Protein preparation

The protein structures of the biological targets were obtained from the RCSB Protein Data Bank (www.rcsb.org) and selected based on their known roles in antioxidant, antibacterial, and antifungal activities. The targets included NADPH oxidase (PDB: 2CDU) and bacterial DNA gyrase subunit B (PDB ID: 3G7E).

Proteins were processed with Maestro’s Protein Preparation Wizard, which involved adding any missing hydrogen atoms and correcting bond orders, especially in regions containing cofactors or coordinated metal ions. Crystallographic water molecules located more than 5 Å away from the active site were removed to avoid interference during docking, while those involved in stabilizing active site geometry were retained. The hydrogen bonding network was optimized, and protonation states of titratable residues like histidine, glutamate, and aspartate were adjusted based on pKa predictions to simulate physiological pH. The proteins were subjected to restrained energy minimization using the OPLS3 force field (Aboul-Soud et al. 2022; Tourabi and Baghouz 2024).

#### Ligand preparation

The docking study began with the preparation of key phytocompounds previously identified in *M. aquatica extracts* through HPLC-ESI-FULL-MS analysis. These compounds were retrieved in SDF format from the PubChem database. The ligands were processed using the LigPrep module within the Maestro software suite (version 11.5, Schrödinger). To ensure accurate 3D representations and energy minimization, the compounds were optimized using the OPLS3 force field. Their ionization states were adjusted to mimic physiological conditions at pH 7.0 ± 2.0. Additionally, to account for stereochemical variability that may influence binding affinity, LigPrep was configured to generate up to 32 stereoisomers per compound (Chebaibi et al. 2024; Taibi et al. 2025).

The detailed rationale for the selection of these specific targets, along with supporting literature, is summarized in Table 1.

**Table 1:** Rationale and references for the selection of in silico protein targets

Target Protein	PDB ID	Rationale for Selection	References
Human NADPH oxidase	2CDU	Key enzyme responsible for the production of reactive oxygen species (ROS). Inhibiting this target is a primary strategy to evaluate the antioxidant potential by preventing radical formation.	(Altenhöfer et al. 2015; Herrera-Calderon et al. 2021)
Gyrase B	3G7E	Essential bacterial enzyme involved in DNA replication, repair, and transcription, making it a well-established target for antibacterial drugs. The GyrB subunit contains the ATP-binding site, making it an attractive target for inhibitors.	(Schoeffler et al. 2010; Collins and Osheroff 2024)

#### Glide standard precision (SP) ligand docking

Molecular docking was conducted using the Standard Precision (SP) mode of the Glide module in Maestro (version 11.5). The receptor grid for each protein was defined around the coordinates of the co-crystallized ligand or the known active site residues. Ligands were docked flexibly, allowing multiple orientations to be sampled. Binding poses were scored based on both binding affinity and geometric complementarity. The conformation with the lowest Glide score was selected as the best pose for each ligand, as it represented the most energetically favorable and structurally plausible binding mode (Beniaich et al. 2022; Tourabi et al. 2023c).

#### Docking validation

To assess the robustness and predictive performance of the molecular docking workflow, a redocking validation strategy was conducted for each selected protein target using its respective co-crystallized ligand. In this procedure, the native ligand was first removed from the crystal structure, subsequently reintroduced into the corresponding active site through the same docking protocol, and the Root Mean Square Deviation (RMSD) between the predicted binding pose and the experimental crystallographic conformation was calculated. An RMSD value lower than 2.0 Å is widely accepted as evidence of a reliable docking setup, reflecting the protocol’s capability to accurately reproduce experimentally observed binding orientations.

The redocking outcomes are summarized below:

- Human NADPH oxidase (PDB ID: 2CDU): Re-docking of the co-crystallized ligand *flavin adenine dinucleotide (FAD)* produced an RMSD of 0.77 Å.
- DNA Gyrase B: Re-docking of the co-crystallized ligand *prop-2-yn-1-yl {[5-(4-piperidin-1-yl-2-pyridin-3-yl-1*,*3-thiazol-5-yl)-1 H-pyrazol-3-yl]methyl}carbamate* generated an RMSD of 1.937 Å.

All calculated RMSD values fall below the commonly accepted threshold of 2.0 Å, thereby demonstrating the stability, precision, and reproducibility of the docking protocol. These findings confirm the suitability of the adopted computational approach for accurately predicting ligand binding conformations within the catalytic sites of the selected protein targets.

### Statistical analysis

All experiments were performed in triplicate, and results are expressed as mean ± standard deviation. Statistical analyses, including One-way ANOVA, were conducted using GraphPad Prism 8.0.2 (263). Additionally, Principal Component Analysis (PCA), correlation coefficient assessment, and polar heatmap visualization were performed using OriginLab Corporation (Northampton, MA, USA).

## Results and discussion

### Preliminary solvent screening

**Fig. 1:**
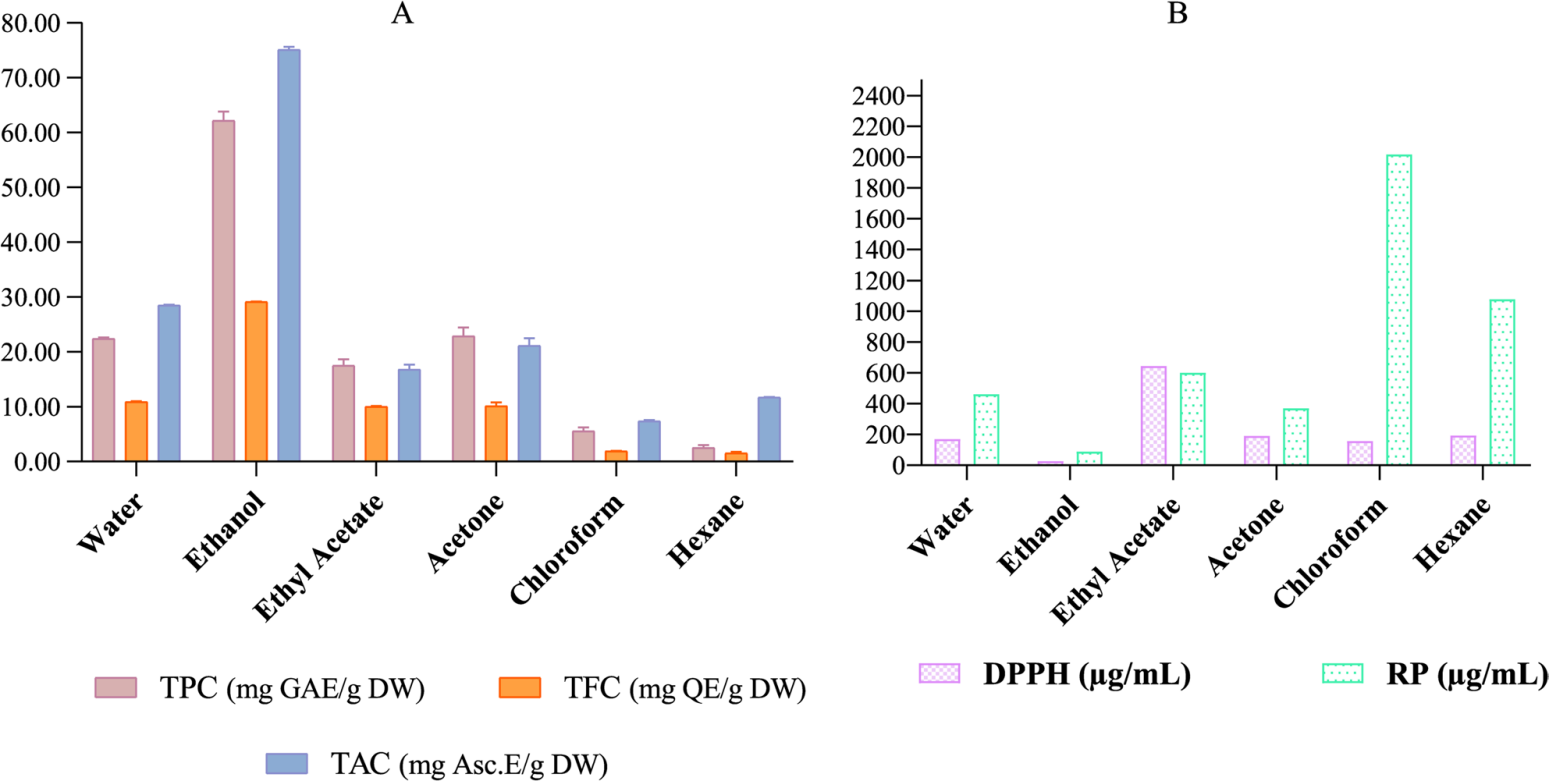
(**A**): Total phenolic amount (TPC) and flavonoid content (TFC), total antioxidant capacity (TAC), (**B**): EC_50_ of reducing power (RP), and IC_50_ of DPPH scavenging activity of different solvents

Choosing the right solvent is a crucial step in phytochemical research. The extraction efficiency depends on several factors such as temperature, solvent polarity, and extraction time. In this study, we conducted an initial evaluation of the solvent extracts to determine their total phenolic content and antioxidant activity (as shown in Fig. 1A and B). We found that hexane, chloroform, and ethyl acetate had minimal impact on antioxidant ability, reductive power, and total antioxidant capacity. These solvents also resulted in lower flavonoid and phenolic contents. Flavonoid and phenolic contents ranged from 1.57 ± 0.20 to 29.16 ± 0.07 mg QE/g DW and 2.56 ± 0.43 to 62.2 ± 1.7 mg GAE/g DW, respectively. Notably, the hydroethanolic extract (ETOH) was optimized to yield higher levels of total phenolic content (TPC) and total flavonoid content (TFC). Our Study indicated that *M. aquatica*’s strong antioxidant capacity, and containing a high concentration of phenolics and flavonoids, was closely linked to the polarity of the solvent, a finding that is confirmatory to our previous study (Tourabi et al. 2023b). In light of these preliminary screening results, we selected the most potent extracts (water, acetone, and 70% ethanol) for further testing of phenolic content and antibacterial activity. The results of this preliminary screening will be presented and discussed in the following section of this study.

### Solvent polarity effect on the extraction yield

**Fig. 2:**
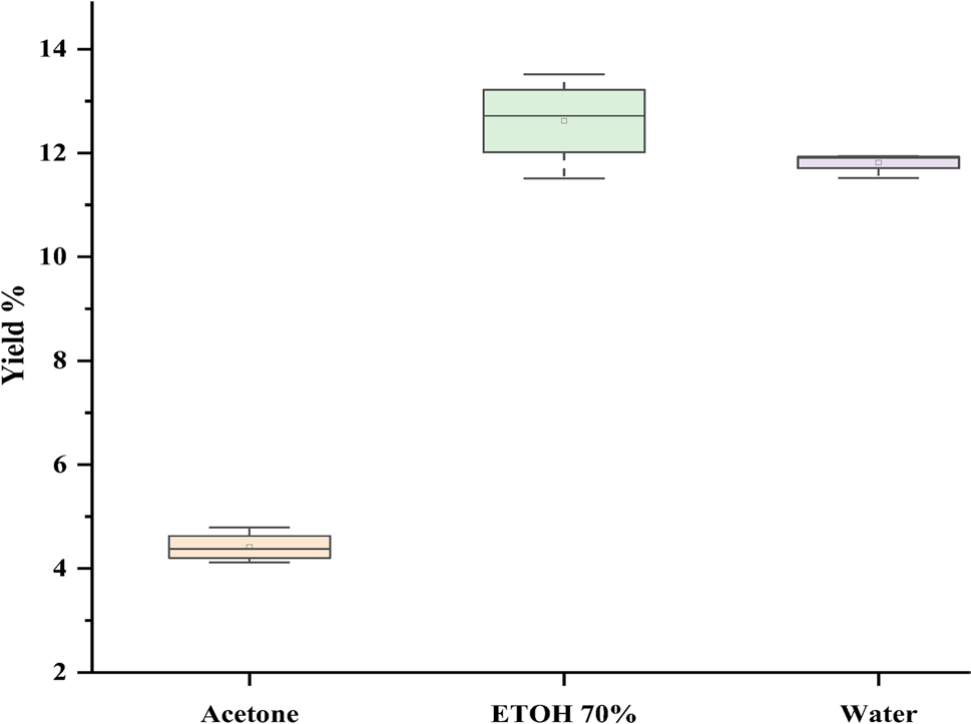
Influence of solvent polarity on the extraction yield of *M. aquatica* extracts. Data are presented as mean ± SD (*n* = 3)

As can be seen in Fig. 2, the hydroethanolic extract has a high yield with a value of 12.51%, followed by the water extract with a level of 11.94%, and the acetone extract had the lowest yield (4.46%). In addition, such clear differentiation also points out the significant effect of polar solvent potency on extraction efficiency rather than the sheer random effect of solvents (Tourabi et al. 2023b). Previous studies indicate a positive relationship between solvents’ polarity and how well they extract chemicals from plant materials, as exemplified in (Lapornik et al. 2005). In this context, it is found that using hydroethanolic and aqueous solutions results in higher yields, which in turn indicates that the major compounds obtained from *M. aquatica* are polar in nature. Moreover, it was ascertained that solvent polarity significantly affects the extraction yield, with polar solvents such as water or a hydroalcoholic mixture producing higher yields than less polar solvents like acetone. It is worth noting that the hydroethanolic solvent performs better due to its intermediate polarity, enabling efficient extraction of both highly and moderately polar compounds (Gil-Martín et al. 2022).

### HPLC-ESI-FULL-MS exploration of individual phenolic composition

The HPLC-ESI-FULL-MS analysis of the aqueous, hydroethanolic, and acetonic extracts of *Mentha aquatica* revealed a distinct qualitative composition depending on the solvent used (Table 2). A total of 18 peaks were tentatively identified based on retention time (RT) and mass-to-charge ratio (m/z). Only a subset of these compounds was detected depending on the solvent system, confirming the differential selectivity and efficiency of each extraction medium.

**Table 2:** Putative annotation of compounds detected by HPLC-ESI-MS based on m/z, RT, and literature comparison

No	RT	m/z (M-H)-	MS Fragment	Proposed compound	Water	EtOH 70%	Acetone
1	2.4	331.1		Galloyl-glucoside	-	-	-
2	5.69	386.7		Tuberonic acid glucoside	-	-	-
3	6.7	308.45		Ferulic acid derivative	-	+	-
4	11.03	376.3		nd	-	+	-
5	12.32	441.28		nd	-	+	-
6	13.72	343.7		2,3,8-Tri-O-methylellagic acid	+	-	-
7	14.01	377.76	312.58	nd	-	+	-
8	16	585.28		3-Hydroxyhispidin-3,4-di-O-glucoside	-	-	-
9	16.31	525.73		nd	-	-	-
10	17.4	248.52	179.15	nd	-	+	-
11	18.15	423.32	372.17	Equisetumpyrone	-	-	+
12	19.08	423.85	377.9	Equisetumpyrone isomer	-	-	+
13	19.88	347.7		Glauconic acid	+	-	-
14	20.07	614.87		nd	-	-	-
15	20.27	395.1		Caffeoyl-protocatechuic acid derivative	-	+	-
16	22.35	301.2	104.12	Quercetin	-	-	+
17	23.35	475.67	377.89	DiosMetin 7-O-beta-D-Glucuronide	-	-	+
18	24.33	471.1	360.86	4’’-Methylepigallocatechin gallate	-	-	-

*RT* Retention time (min), *nd* Non-detected

**Fig. 3:**
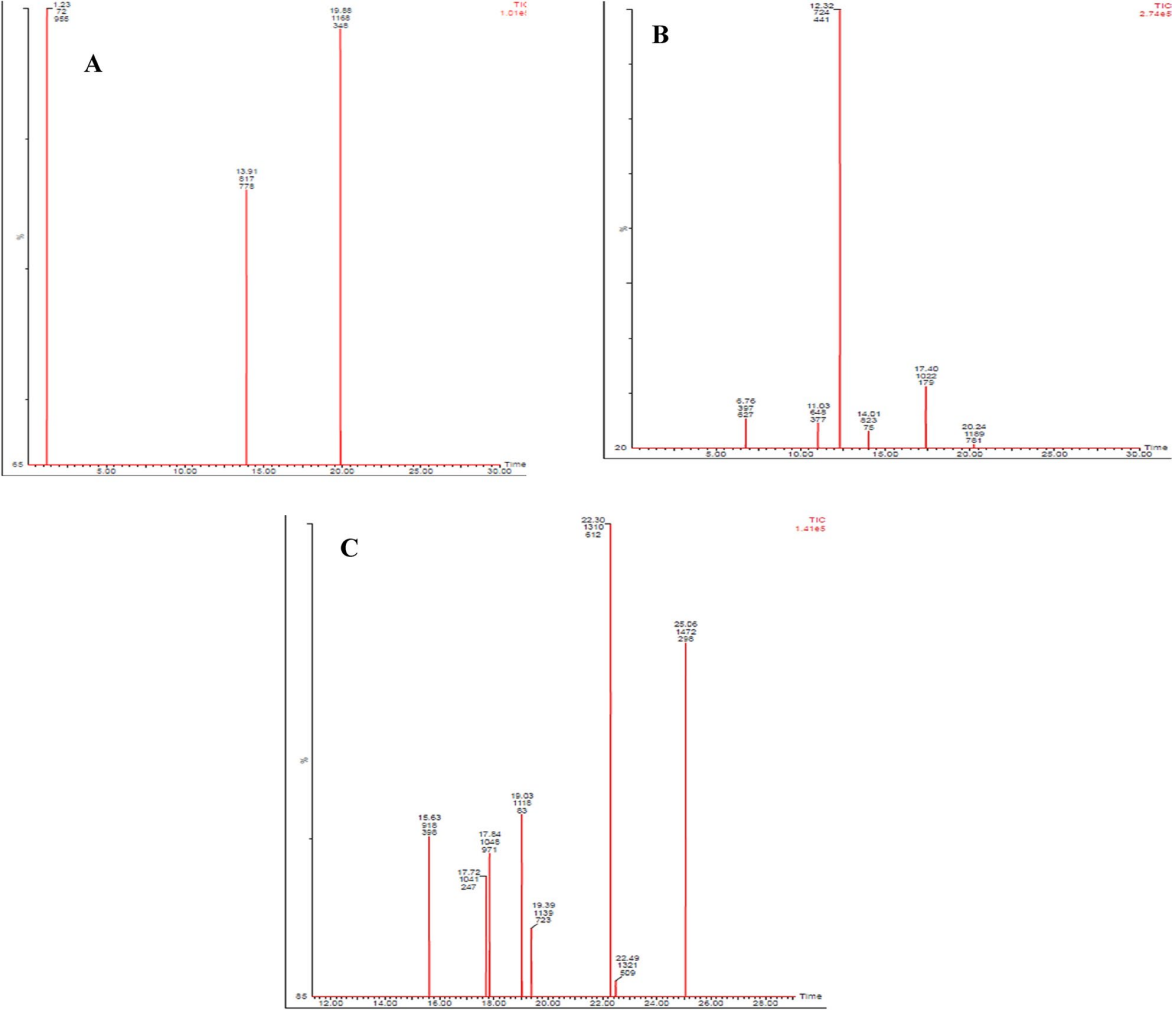
LC-MS chromatograms of *M. aquatica* obtained from various solvent extracts: (**A**) aqueous extract, (**B**) hydroethanolic extract, and (**C**) acetone extract

Among the detected compounds, 2,3,8-Tri-O-methylellagic acid (RT: 13.72 min; m/z: 343.7) and Glauconic acid (RT: 19.88 min; m/z: 347.7) were detected only in the aqueous extract under our LC-MS analytical conditions. This observation suggests that water may favor the extraction of more polar constituents (Fig. 3A). This result is consistent with the high polarity of these acids and aligns with previous findings by Tourabi et al., where aqueous extracts showed a predominance of hydrophilic phytochemicals, particularly those bearing polar functional groups (Tourabi et al. 2023b, 2025).

The hydroethanolic extract showed the richest chemical diversity, including several unidentified compounds (RT 6.70, 11.03, 12.32, 14.01, 17.40, 20.27) and a ferulic acid derivative (RT: 6.70 min; m/z: 308.45) (Fig. 3B), consistent with previous reports describing hydroethanol (EtOH 70%) as the most efficient solvent system for extracting both hydrophilic and lipophilic phenolics. Additionally, the chemical diversity further suggests that hydroethanol is a balanced solvent system which combines the abilities of both water and ethanol in enhancing the extraction of compounds with diverse polarities. The detection of ferulic acid derivatives is particularly relevant given their recognized antioxidant and antibacterial properties, also highlighted in in silico analyses by Tourabi et al., where ferulic acid was one of the main contributors to antioxidant activity (Tourabi et al. 2023b).

The acetonic extract was the only one where Equisetumpyrone and its isomer (RT: 18.15 and 19.08 min) were detected, suggesting the solvent’s ability to extract less polar, lipophilic constituents (Fig. 3C). Furthermore, quercetin (RT: 22.35 min; m/z: 301.2), a well-known flavonols with potent bioactivities, was exclusively identified in the acetonic extract, aligning with earlier studies where acetone-based systems extracted high levels of flavonoids, this can be attributed to its moderate polarity and ability to penetrate the plant matrix, which enhances solubilization of less polar flavonoid aglycones and aromatic phenolics that are poorly extracted by highly polar solvents such as water (Herrera-Rocha et al. 2022a). Our observations confirm that different solvents, due to their unique chemical properties like polarity, solubility, and affinity for specific components, can extract a diverse array of phytochemicals from the same source material.

Thus, we have searched recent publications using HPLC/LC-MS to characterize phenolic compounds in *M. aquatica* or closely related *Mentha* species. Several studies report phenolic identifications in *M. aquatica* (Lahlou et al. 2024), or in other species with comparable profiles (Ćavar Zeljković et al. 2021; Esmaeili et al. 2025).

### Total phenolic and flavonoid content

**Fig. 4:**
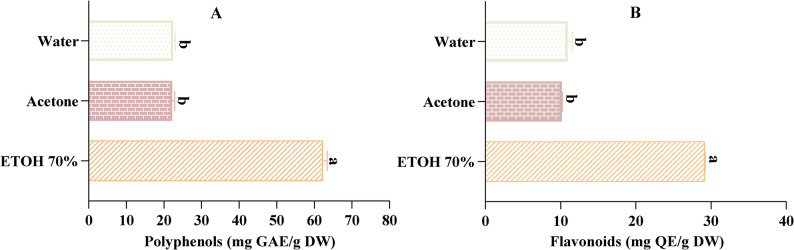
Total phenolic content (**A**) and total flavonoid content (**B**) of selected solvent extracts of *M. aquatica*. Values are presented as mean ± SD (*n* = 3). Within each assay, values sharing the same letter are not significantly different (*p* > 0.05)

Secondary metabolites produced by plants, such as alkaloids, terpenoids, and phenolic compounds, are among the most prevalent categories of natural products. Notably, coumarins, phenolic acids, condensed tannins, and hydrolyzable, as well as flavonoids, are extensively consumed as medicine and as nutrients (Naczk and Shahidi 2006). Furthermore, phenolic compounds possess important bioactivity, namely antibacterial, antioxidant, anti-inflammatory, anti-infective, and antiproliferative agents (Masibo and He 2008). Fig. 4 (A and B) illustrates the measured total phenolic content (TPC) and total flavonoid content (TFC) of the analyzed samples. The hydroethanolic extract achieved the best TPC at about 62.2 ± 1.2 mg GAE/g DW, followed by the water extract at 22.4 ± 0.6 mg GAE/g DW, and then the acetone extract at 22.2 ± 0.6 mg GAE/g DW (Fig. 4A). Our study’s findings indicate that polar solvents can extract more than just phenolic compounds more effectively than non-polar solvents, and our results exceed those reported by Abbas and his colleagues (Abbas et al. 2021). Conversely, Dorman et al., found that the water extract had a notably great phenolic tenor, measuring 152.5 mg GAE/g DW (Dorman et al. 2003). Other research by Hoai et al. (Hoai et al. 2023) reported a higher TPC in the 50% acetone extract, with a value of 120.92 mg GAE/g DW.

Figure 4B displays the results for the total flavonoid amount. The high flavonoid concentration was found in the hydroethanolic extract (EtOH, 70%), measuring 29.15 ± 0.09 mg QE/g DW. In comparison, the acetonic extract had a flavonoid content of 10.17 ± 0.07 mg QE/g DW, while the water extract was quantified at 10.9 ± 0.6 mg QE/g DW. Such a distinct variation also underscores the importance of solvents, which are essential for flavonoid extraction. The hydroethanolic extract yields more flavonoids due to its intermediate polarity, which enables the extraction of both aglycones and glycosides. Water and acetone, however, seem to facilitate more specific extraction of highly polar and less polar flavonoids, respectively, yielding fewer flavonoids. These findings are in line with the reports respectively published by Tourabi et al. (Tourabi et al. 2023b), which report that the hydroethanolic extract of *M. longifolia* has multiple hydroxyl or carboxyl groups, which enhance their solubility in polar solvents. In another related study, Abbas et al., found that the hydroethanolic fraction of *Curcuma longa* and *Mentha aquatica* had the highest content of flavonoids and phenolic compounds compared to their hydro-methanolic and hydro-organic fractions. The reason behind their finding could be related to the moderately polar nature of hydro-ethanol solution, which enables it to dissolve more flavonoid aglycones and moderately polar flavonoid glycosides for higher antioxidant and antimicrobial activities (Abbas et al. 2021). This discrepancy can be accounted for by the diversity in the solvent, plant part, and geographical location, as these factors are also known to affect the phenolic content. Besides, flavonoids and phenolic metabolites have been identified as compounds with several therapeutic properties, including anti-inflammatory, antibacterial, antioxidant, and anti-cancer effects (Tungmunnithum et al. 2018). Since the hydroethanolic extract provides a higher yield, it may be the most effective extraction solvent for use in dietary supplements, industrial applications, or as an active ingredient in natural medicines. Acetone or other organic solvents were preferred in some earlier experiments, but this result differs from that.

### Antioxidant activity

**Fig. 5:**
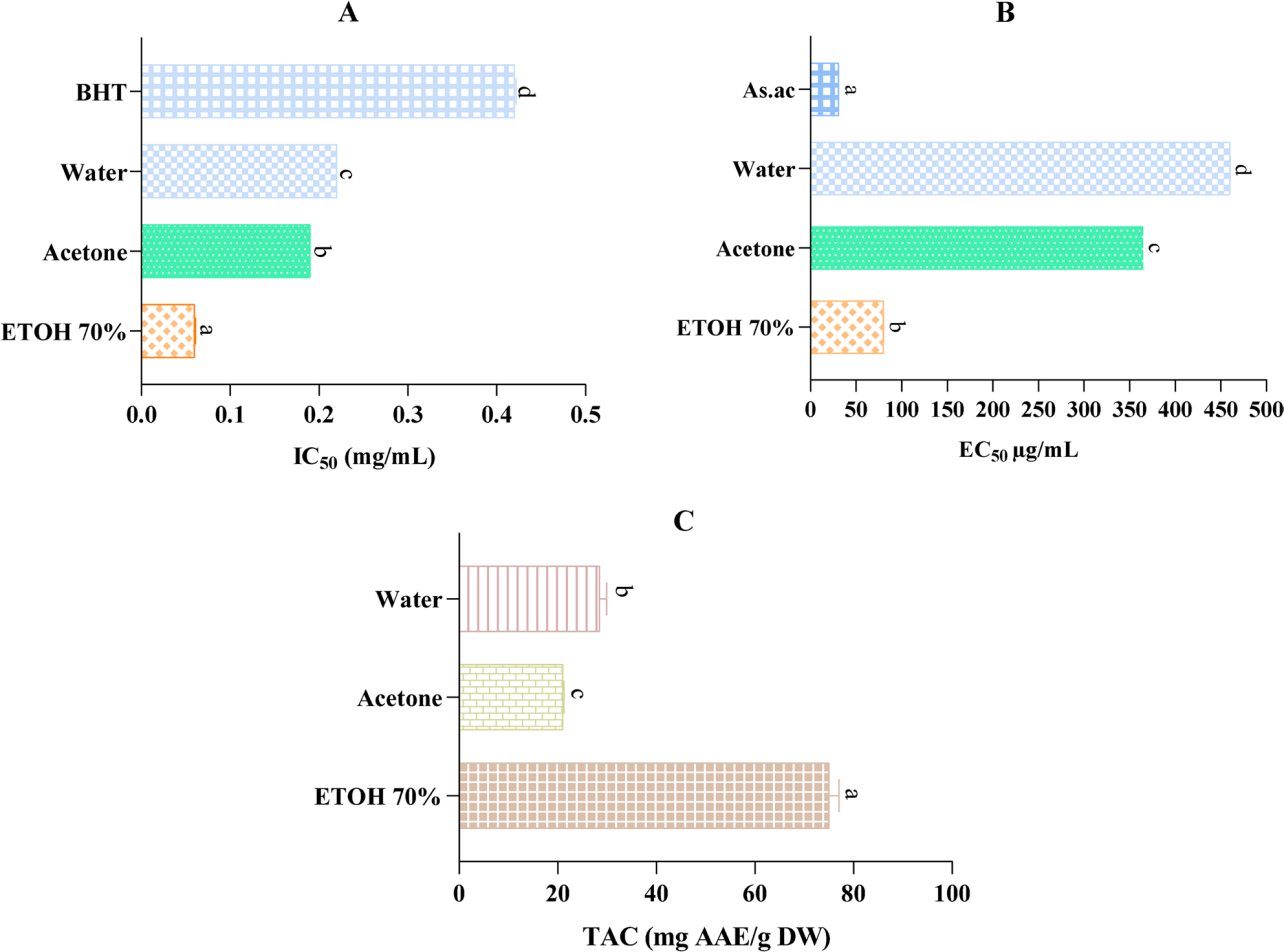
Antioxidant properties of *M. aquatica* extracts: (**A**) IC₅₀ values determined by the DPPH assay, (**B**) EC₅₀ values for the reducing power assay, and (**C**) total antioxidant capacity. Values sharing the same letter within a given assay do not differ significantly (*p* > 0.05). Data are presented as mean ± SD (*n* = 3). BHT: butylated hydroxytoluene; As.ac: ascorbic acid

The antioxidant activity was assessed using the DPPH radical scavenging assay, the ferric-reducing power assay, and the measurement of total antioxidant capacity. The data are depicted in Figs. 5A, B, and C. These outcomes demonstrated notable differences between the various extracts. The high EC_50_ of 80 µg/mL for the RP test and IC_50_ values of 0.06 ± 0.001 mg/mL for the DPPH radical suggest that the hydroethanolic extract demonstrated excellent antiradical capacity and reducing power. With an IC_50_ value of 0.19 ± 0.02 mg/mL and an EC_50_ value of 370 µg/mL, respectively, the acetonic extract displayed the greatest values (Fig. 5A and B). Furthermore, Fig. 5C shows that the overall antioxidant potential of the acetonic extract was 21.1 ± 0.1 mg AAE/g DW, while the hydroethanolic extract had a greater total antioxidant capacity of 75 ± 2.0 mg AAE/g DW, followed by the water extract with 28.5 ± 1.0 mg AAE/g DW. The findings by Thi et al. (Thi et al. 2020) showed that hydroethanolic and water extracts of *M. aquatica* had lower antioxidant capacities with IC_50_ values of 306.97 ± 13.78 and 142.98 ± 19.93 µg/mL, respectively, in contrast with those of Conforti et al. (Conforti et al. 2008), who reported a low IC_50_ value of 29 µg/mL for the hydroethanolic extract’s scavenging activity. Our results indicate that the hydroalcoholic extract is particularly effective for antioxidant activity. In addition, solvents like ethanol/methanol are recognized for their abilities to extract hydrophilic and liposoluble phenolic compounds such as phenolic acids (e.g., gallic acid and caffeic acid), due to their intermediate polarity and strong hydrogen-bonding capacity. Thus, hydroethanolic extract could maximize the recovery of these compounds, which is crucial for antioxidant capacities. This result is all the more important as it could allow for the development of more effective natural extracts to combat oxidative stress in the fields of health or cosmetics. The ANOVA analysis indicated a statistically significant variation (*p* < 0.05) in the efficacy of the three solvents employed for extracting compounds from *M. aquatica* leaves. Our finding aligns with previous research, which indicates that highly polar solvents such as water, ethanol, methanol, as well as acetone are typically the most efficient for extracting antioxidant compounds. The variability in antioxidant capacity is often due to the synergistic effects of such phytochemical components. For instance, the FRAP assay conducted by Hajimehdipoor and colleagues demonstrated that the enhanced antioxidant potential of combinations, such as combinations involving quercetin with gallic acid and caffeic acid, or quercetin with gallic acid and rutin (Hajimehdipoor et al. 2013). Moreover, the antioxidant activity is mainly due to phenolic acids, flavonoids, terpenoids, and their interactions.

### Assessment of the antibacterial capacity

The data shown in Table 3 demonstrate the solvent extract from *M. aquatica’s* strong antibacterial capabilities against a variety of bacterial cultures, such as *E. Coli*,* P. aeruginosa*,* B. cereus*, and *S. aureus.* More particularly, the acetonic extract demonstrated a high inhibitory concentration against *B. cereus* bacteria (MIC = 0.8 ± 0.1 mg/mL and MBC = 0.8 ± 0.3 mg/mL) as well as *S. aureus* (MIC = 1.6 ± 0.1 mg/mL and MBC = 1.6 ± 0.3 mg/mL). Similarly, the acetone extract demonstrated significant antibacterial activity against *E. coli*, with a MIC of 4.7 ± 0.0 mg/mL. Moreover, the hydroethanolic extract showed strong inhibitory effects against *B. cereus*, with a MIC of 1.8 ± 0.6 mg/mL. Antibiotics used as positive controls (gentamycin and kanamycin) displayed much lower MICs and MBCs, indicating higher potency (Table 3). Notably, both acetone and hydroethanolic extracts exhibited higher antibacterial efficacy against Gram-positive bacteria compared to Gram-negative strains. These observations are in agreement with the findings reported by (Tourabi et al. 2023b), which reported that the acetone extract of *M. longifolia* exhibited pronounced antibacterial activity, with particularly strong effects against *Staphylococcus aureus* and *Bacillus cereus*. In another study performed by Ferhat et al. (Ferhat et al. 2017), the antibacterial activity of the chloroform and methanolic extracts from the aerial parts of *M. aquatica* was evaluated against a panel of multidrug-resistant bacteria. The results revealed that the methanolic extract exhibited strong antibacterial potential, with a minimum inhibitory concentration (MIC) of 0.128 ± 1.30 mg/mL. Notably, all tested extracts, except the hydroethanolic extract, demonstrated a minimum bactericidal concentration (MBC) to MIC ratio (MBC/MIC) below 4, suggesting a pronounced bactericidal effect against *B. cereus* and *S. aureus*. This interpretation aligns with the established criterion that compounds with an MBC/MIC ratio ≤ 4 are generally considered bactericidal, whereas those with a ratio > 4 tend to act bacteriostatically (Konaté et al. 2012).

**Table 3:** Minimum inhibitory concentrations (MIC) and minimum bactericidal concentrations (MBC) of *Mentha aquatica* extracts expressed in mg/mL

Bacterial strains	EtOH 70%				Acetone				Water				Kanamycin				Gentamycin			
	MIC	MBC	MBC/MIC	Effect	MIC	MBC	MBC/MIC	Effect	MIC	MBC	MBC/MIC	Effect	MIC	MBC	MBC/MIC	Effect	MIC	MBC	MBC/MIC	Effect
Gram-negative Bacteria																				
* P. aeruginosa*	6.3 ± 0.0	ND			6.3 ± 0.0	ND			6.4 ± 0.2	ND							0.3 ± 0.5	0.7 ± 0.2	2	Bactericidal
* E. coli*	6.3 ± 0.1	ND			4.7 ± 0.0	ND			12.6 ± 0.0	ND							0.016 ± 0.0	0.032 ± 0.1	2	Bactericidal
Gram-positive Bacteria																				
* S. aureus*	6.2 ± 0.1	50 ± 2	8.1 ± 0.4	Bacteriostatic	1.6 ± 0.1	1.6 ± 0.3	1	Bactericidal	6.3 ± 0.1	6 ± 1	1 ± 0.0	Bactericidal	0.016 ± 0.1	0.032 ± 0.0	2	Bactericidal				
* B. cereus*	1.8 ± 0.6	50 ± 1	28.1 ± 0.0	Bacteriostatic	0.8 ± 0.1	0.8 ± 0.3	1	Bactericidal	3.1 ± 0.1	25 ± 0.0	8.1 ± 0.2	Bacteriostatic	0.008 ± 0.2	0.008 ± 0.7	1	Bactericidal				

*ND* Non-determined, *MBC* Minimal bactericide concentration, *MIC* Minimal inhibitory concentration

Our experimental results indicate that Gram-positive bacteria were more sensitive to the tested extracts than Gram-negative strains. Mechanistically, the intricate double-membrane structure of Gram-negative bacteria, comprising a lipoprotein layer and a lipopolysaccharide (LPS) component of the cell envelope, likely contributes to their decreased susceptibility (Janakat et al. 2015). Additionally, the strong antibacterial activity of *M. aquatica* leaf extracts can be attributed to their high content of bioactive phenolic acids and flavonoids (Table 1), as compounds such as ferulic acid, quercetin, and their derivatives have previously demonstrated notable antibacterial effects (Gutiérrez-del-Río et al. 2018; Adamczak et al. 2020).

As was previously reported, phenolic compounds have been extracted from plant material with success using a variety of solvent combinations. The most widely used solvents are acetone, water, methanol, and ethanol in their aqueous mixtures (Michiels et al. 2012). This may be explained by the superior ability of acetone to solubilize highly polar bioactive compounds, including quercetin and diosmetin 7-O-beta-D-glucuronide, which can target bacterial membranes (Chan et al. 2013; Wang et al. 2018; Malczak and Gajda 2023). Interestingly, the acetonic extract showed the ultimate antibacterial effect, particularly against Gram-positive bacteria. The fact that the acetonic extract is particularly effective against Gram-positive bacteria is crucial, as it could have applications in the fight against resistant bacterial infections. This result may be unexpected, as some studies have focused more on ethanol for antibacterial activity, but in our experimentation, acetone shows stronger efficiency.

Acetonic and hydroethanolic extracts were the most effective at extracting phytochemicals with a wide range of biological characteristics (Herrera-Rocha et al. 2022b). These phytochemicals have shown strong antibacterial activity against multi-resistant bacteria through several mechanisms of action, including targeting metabolites produced by bacteria, disrupting membranes and cell walls, influencing lipid membranes, interacting with ion channels and membrane receptors, blocking the synthesis of ATP and enzymes, and stopping the formation of biofilms, which represent some of these ways. It has also been demonstrated that some polyphenol and antibiotic combinations have synergistic effects (Álvarez-Martínez et al. 2020). Gallic acid, as shown by Campos et al., may help modulate the hydrophilic nature of pathogens through interaction with the surfaces of both Gram-positive and Gram-negative bacteria. This mechanism can promote potassium ion efflux and trigger protein denaturation, which could result in an acidification of the pathogens’ cytoplasm. These impacts have the potential to alter the cytoplasmic membrane’s pliability, cause intracellular materials to be expelled, and destroy membranes of pathogens (Campos et al. 2009). Other trials have revealed high antibacterial activity of the flavonols quercetin and kaempferol against *S. aureus*, with MICs value of 0.00195 mg/mL and 0.0078 mg/mL, respectively (Su et al. 2014; Mokhtar et al. 2017). Studies indicate that naringenin can inhibit multiple *Staphylococcus aureus* strains, producing low-level antibacterial effects with MICs range from 0.256 to 0.512 mg/mL (Zhang et al. 2013).

### Molecular docking assessment

Although the extract demonstrated significant chemical antioxidant activity in DPPH, RP, and TAC assays, these methods primarily evaluate radical scavenging and reducing capacity rather than direct enzymatic inhibition. To further explore possible molecular interactions, an in-silico docking study was performed against NADPH oxidase activity, a key enzyme that reduces intracellular ROS levels, thereby limiting oxidative stress and preventing associated cellular damage. This mechanism plays a crucial role in maintaining redox balance and protecting biological systems against oxidative injury. In our in-silico study, diosmetin 7-O-beta-D-glucuronide, quercetin, and equisetumpyrone exhibited the strongest inhibition against NADPH oxidase with glide gscore of -6.828, -6.587, and − 6.002 kcal/mol.

DNA gyrase, a crucial bacterial enzyme, consists of two functional subunits, GyrA and GyrB, and is fundamental in the process of DNA replication through its ability to introduce negative supercoils into DNA strands. The GyrB subunit facilitates ATP binding and hydrolysis, supplying the energy necessary for supercoiling activity. Targeting and inhibiting GyrB effectively prevents ATP hydrolysis, impairing the entire gyrase function. This results in a breakdown of DNA replication and transcription processes, ultimately leading to bacterial cell death. Based on our in silico docking analysis, the compounds quercetin, 2,3,8-Tri-O-methylellagic acid, and diosmetin 7-O-beta-D-glucuronide exhibited the strongest affinity toward GyrB, with glide gscores of -7.023, -6.827, and − 5.888 kcal/mol, respectively (Table 4).

**Table 4:** Docking results of various ligands across multiple receptor targets

	NADPH oxidase (PDB: 2CDU)	Gyrase B (PDB: 3G7E)
2,3,8-Tri-O-methylellagic acid	-4.254	-6.827
DiosMetin 7-O-beta-D-Glucuronide	-6.828	-5.888
Equisetumpyrone	-6.002	-4.939
Ferulic acid derivative	-5.401	-5.253
Glauconic acid	-5.362	-3.506
Quercetin	-6.587	-7.023

**Fig. 6:**
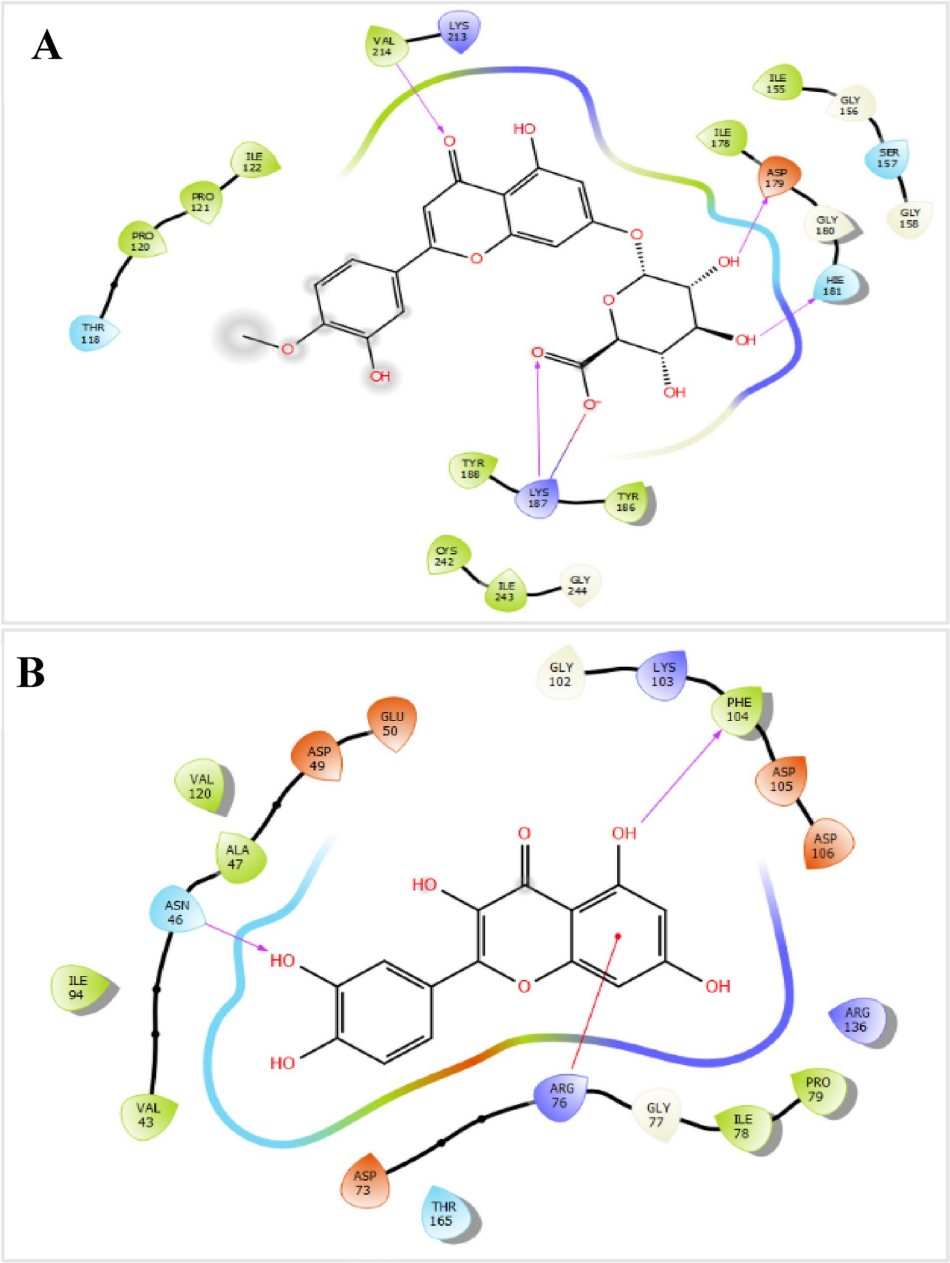
Two-dimensional viewer of ligand interactions within the active sites of target proteins. (**A** ): DiosMetin 7-O-beta-D-Glucuronide interaction with NADPH oxidase active sites. (**B**) Quercetin liaison with energetic site of active site of gyrase B

**Fig. 7:**
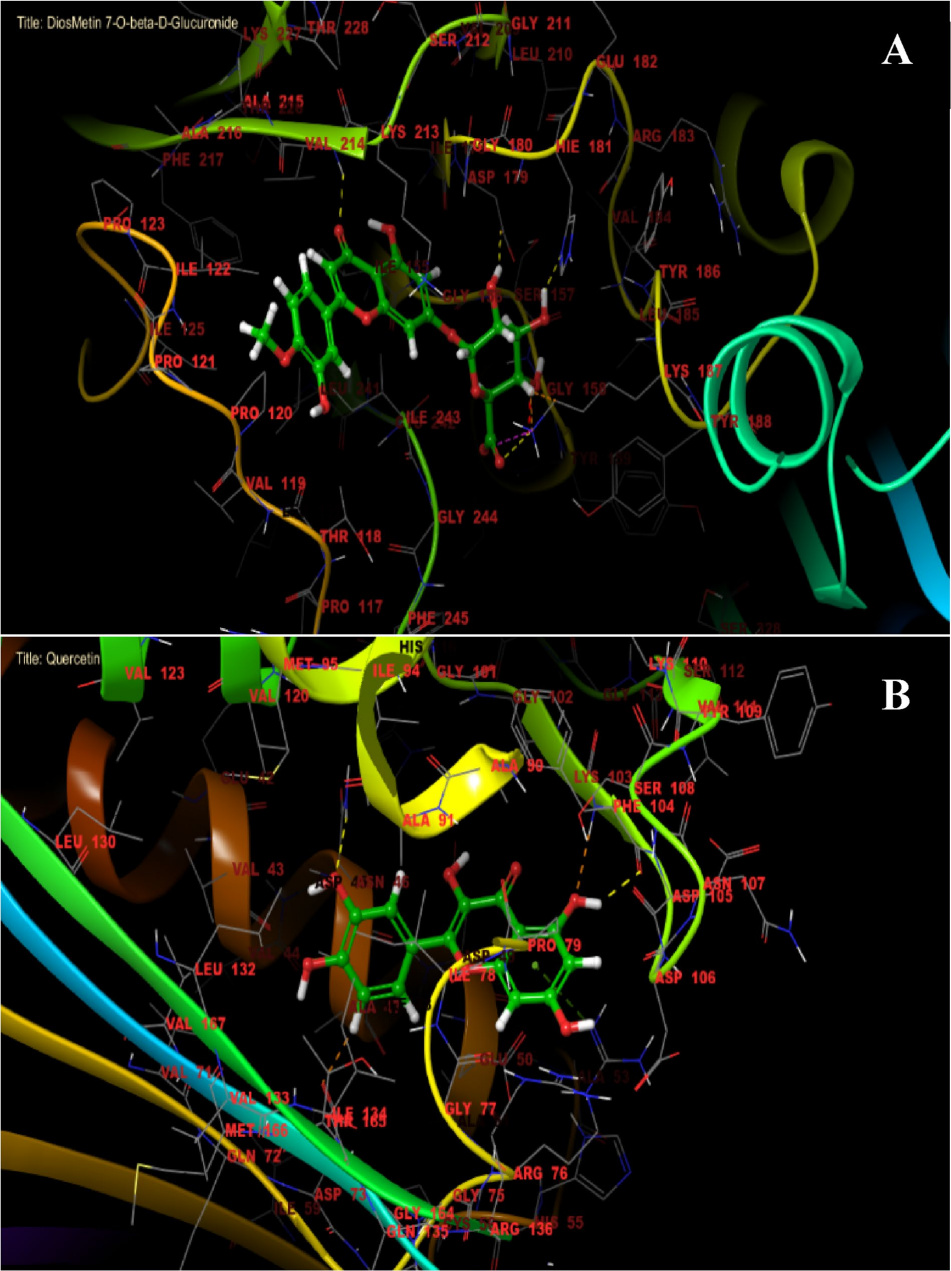
Three-dimensional viewer of ligand liaison within the active sites of target proteins. (**A**): DiosMetin 7-O-beta-D-Glucuronide interaction with NADPH oxidase active sites. (**B**) Quercetin interaction with the active site of active site of gyrase B

The 2D and 3D interaction analyses showed that diosmetin 7-O-beta-D-glucuronide formed four hydrogen bonds with residues VAL 214, ASP 179, HIE 181, LYS 187, and one salt bridge with residue LYS 187 within the active site of NADPH oxidase (Fig. 6A and 7A). Although Quercetin was identified as the most active antibacterial compound, it forms two hydrogen bonds with the ASN46 residue and PHE 104, and one Pi cation bond with residue ARG 76 in the active site of Gyrase B (Figs. 6B and 7B).

### Statistical analysis

#### Correlation analysis

**Fig. 8:**
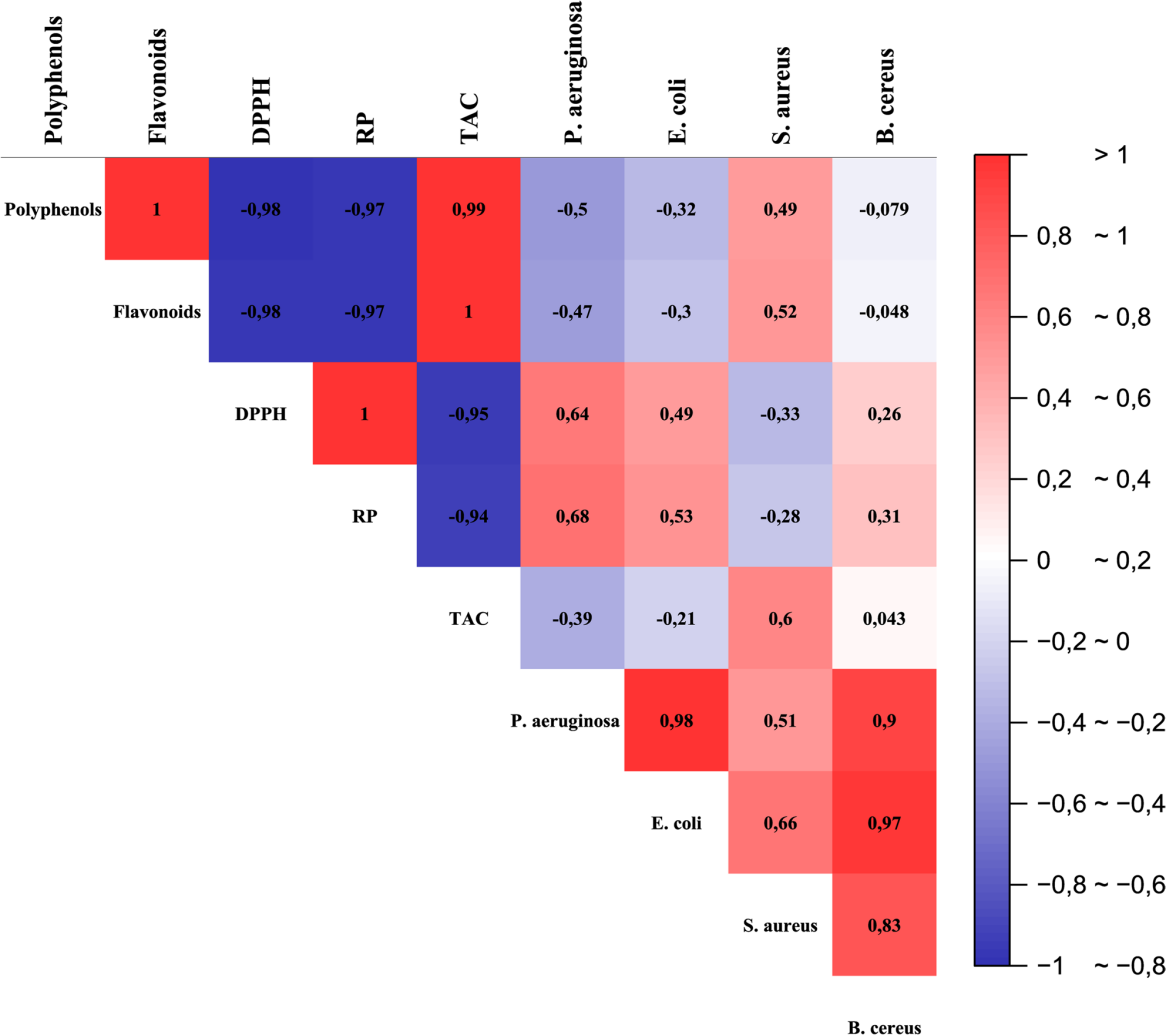
Pearson correlation analysis between the antioxidant components (polyphenols and flavonoids), DPPH radical scavenging activity, reducing power (RP), total antioxidant capacity (TAC), and antibacterial activity of *M. aquatica* solvent extracts. The strength and direction of the correlations are represented by different square colors

A correlation test is considered a valuable method for identifying relationships among the various components under study. In our current investigation, we reevaluate the relationship between antioxidant potential and its quantity using the average antioxidant importance of all solvent extracts examined, as well as the relation between antioxidant tenor and antibacterial efficacy. Fig. 8 illustrates the findings obtained from the correlation assay. We found a significant positive correlation between the phenolic content, flavonoids, and total antioxidant capacity (TAC), with correlation coefficients of r²=1 and r²=0.99. However, a notable negative correlation was observed between phenolic components, flavonoids, and antioxidant capacity, with correlation coefficients of r²=-0.98 and r²=-0.97, respectively, as evaluated by DPPH and RP tests. This negative correlation reflects the fact that these assays report activity as IC₅₀ values, where a lower IC₅₀ indicates stronger antioxidant activity. Therefore, the negative correlation actually indicates that extracts with higher phenolic and flavonoid contents exhibit stronger antioxidant capacity. Furthermore, there was little correlation between the polyphenolic and flavonoid contents as well as the antibacterial effectiveness of the *M*. *aquatica* extracts. The results obtained in this study are consistent with previous reports, which highlight a strong association between phenolic content, antioxidant activity, and antibacterial efficacy (Matejczyk et al. 2018; Tourabi et al. 2023b).

#### Polar correlation heatmap

**Fig. 9:**
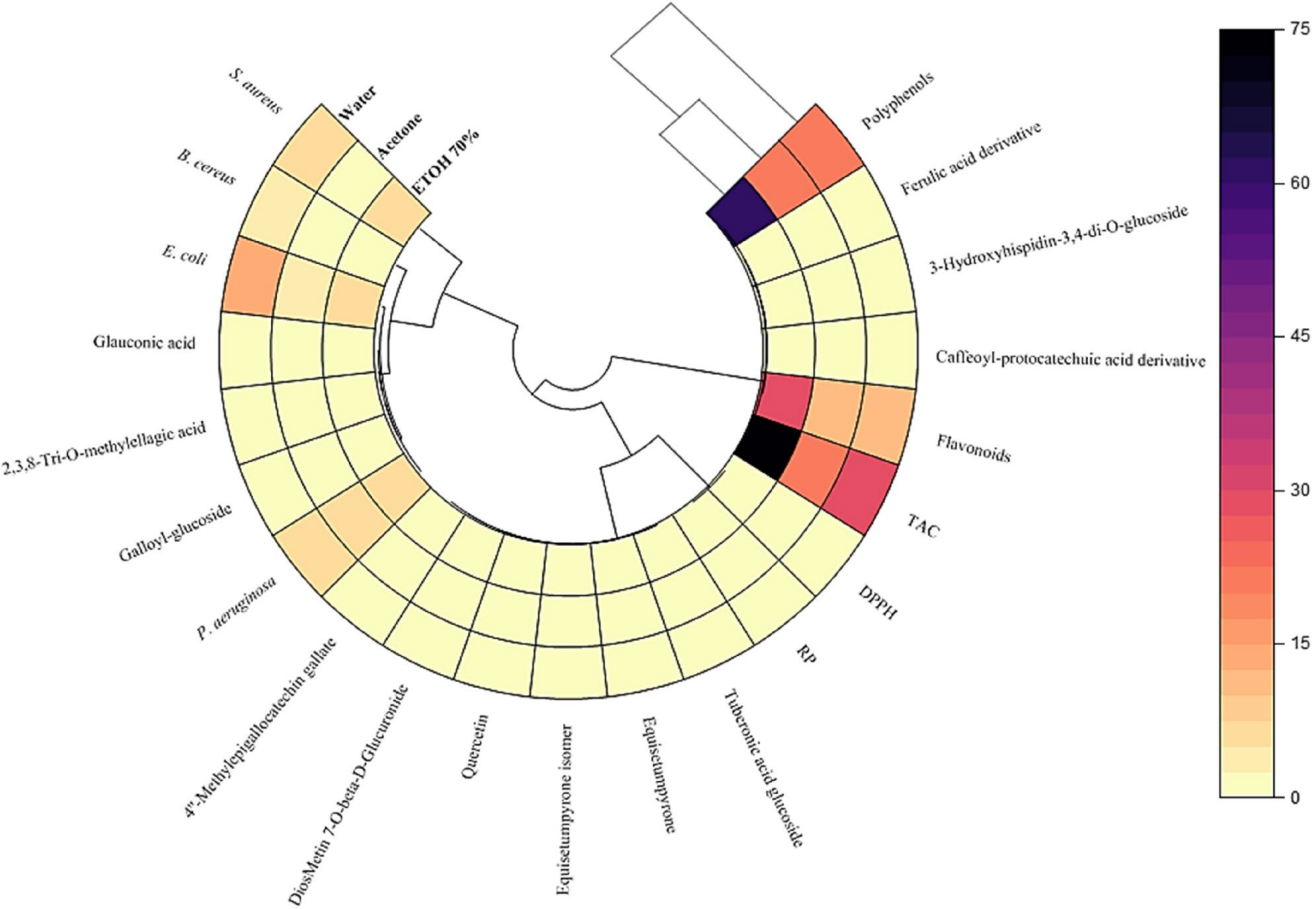
Circular dendrogram overlaid on a polar heatmap illustrating the correlations among individual components, polyphenols, flavonoids, and their antioxidant and antibacterial activities in the solvent extract

Figure 9 presents a polar heatmap illustrating the correlations among individual phenolic compounds, total phenolic and flavonoid content, as well as antioxidant and antibacterial activities. In this representation, lower values are indicated in yellow, while higher values are depicted in black. The polar heatmap analysis made it challenging to cluster samples based on overall similarity. Notably, the solvent extracts were divided into two distinct groups: one consisting of the 70% ethanol extract and the other comprising the water and acetone extracts. Furthermore, the heatmap revealed clear correlations between individual phenolic constituents, total phenolic and flavonoid contents, and the measured antioxidant and antibacterial activities.

#### Principal component analysis (PCA)

**Fig. 10:**
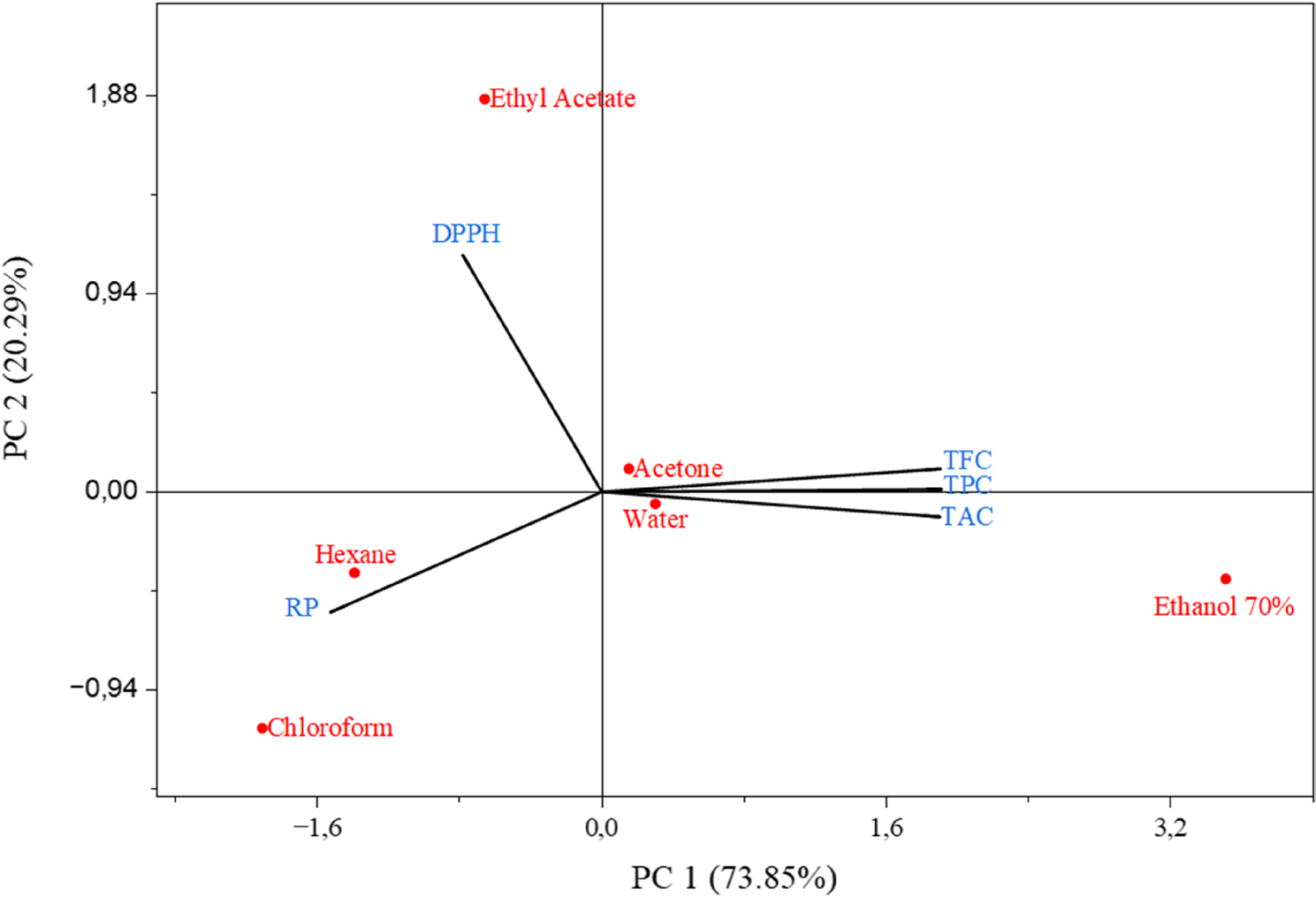
Principal component analysis (PCA). Charts showing the antioxidant properties and phenolic components in the solvent extracts under study

Principal Component Analysis (PCA) was conducted to interpret the previously collected data, and the results are illustrated in Fig. 10. Specifically, Fig. 10 presents the PCA results for total phenolic content (TPC), total flavonoid content (TFC), individual phenolic compounds, and the antioxidant activities of the solvent extracts. The analysis revealed that the first two principal components (PC1 and PC2) captured the entirety of the variance (100%) among the selected variables. PC1 was mainly characterized by the positive loadings of TPC, TFC, and TAC, which were grouped together in a tight cluster along the positive side, suggesting a positive correlation among these variables. This implies that the phenolic and flavonoid contents are important factors that contribute to the variation described by PC1 and are closely related to total antioxidant capacity. DPPH and RP, on the other hand, were positioned in opposite directions in the biplot, suggesting a different pattern of variation compared to the phenolic-related variables. This implies that the two assays may measure different aspects of antioxidant activity.

The position of the extracts in the plot further supports the above interpretation. The 70% ethanol extract was positioned on the positive side of PC1, close to TPC, TFC, and TAC, suggesting a relative relationship with higher amounts of these variables. The water and acetone extracts were positioned close to the origin, suggesting an intermediate position. The hexane and chloroform extracts, on the other hand, were positioned on the negative side of PC1, suggesting a weaker relationship with the phenolic-related variables. Ethyl acetate was mainly separated along PC2, suggesting differentiation based on variables that contribute to this component, mainly DPPH.

## Conclusion

This study provides new insights into the influence of solvent polarity on antioxidant properties, antibacterial efficiency, and phenolic profile of *Mentha aquatica* leaves, with findings that stand out from previous research in several key ways. First, the largest amount of flavonoids and phenolic compounds mentioned in the hydroethanolic extract illustrates the plant’s diverse bioactive profile, which has not received enough attention in prior studies. By employing various solvents, we demonstrated a clear relation between solvent polarity and the extraction efficiency of bioactive compounds. Specifically, hydro-ethanol was identified as the most effective solvent to maximize antioxidant activity, contrary to previous studies that often favored organic solvents such as acetone a key contribution of this research is the comparative examination of antibacterial activity of different solvents with different polarities, where the acetone extract has shown remarkable efficacy, particularly against Gram-positive bacteria, which stand *M. aquatica* as a powerful source of new antibacterial agents. This finding sets our study apart, highlighting the potential of acetonic extracts from *M. aquatica* as a potent antibacterial agent, an area that has received limited attention in prior research. Moreover, the dual activity of these antioxidant and antibacterial extracts suggests their potential use as multifaceted, incorporated in foods as preservatives, nutraceuticals, or pharmaceutical industries.

## Data Availability

All data generated or analyzed during this study are included in this published article.
